# Evaluating the Process of Online Health Information Searching: A Qualitative Approach to Exploring Consumer Perspectives

**DOI:** 10.2196/jmir.3341

**Published:** 2014-10-07

**Authors:** Alexander S Fiksdal, Ashok Kumbamu, Ashutosh S Jadhav, Cristian Cocos, Laurie A Nelsen, Jyotishman Pathak, Jennifer B McCormick

**Affiliations:** ^1^Mayo ClinicBiomedical Ethics ProgramRochester, MNUnited States; ^2^Mayo ClinicBiomedical Statistics and InformaticsRochester, MNUnited States; ^3^Mayo ClinicGlobal Business SolutionsRochester, MNUnited States; ^4^Mayo ClinicGeneral Internal MedicineRochester, MNUnited States; ^5^Mayo ClinicHealth Care Policy and ResearchRochester, MNUnited States

**Keywords:** Internet, information seeking behavior, consumer health information, qualitative research

## Abstract

**Background:**

The Internet is a common resource that patients and consumers use to access health-related information. Multiple practical, cultural, and socioeconomic factors influence why, when, and how people utilize this tool. Improving the delivery of health-related information necessitates a thorough understanding of users’ searching-related needs, preferences, and experiences. Although a wide body of quantitative research examining search behavior exists, qualitative approaches have been under-utilized and provide unique perspectives that may prove useful in improving the delivery of health information over the Internet.

**Objective:**

We conducted this study to gain a deeper understanding of online health-searching behavior in order to inform future developments of personalizing information searching and content delivery.

**Methods:**

We completed three focus groups with adult residents of Olmsted County, Minnesota, which explored perceptions of online health information searching. Participants were recruited through flyers and classifieds advertisements posted throughout the community. We audio-recorded and transcribed all focus groups, and analyzed data using standard qualitative methods.

**Results:**

Almost all participants reported using the Internet to gather health information. They described a common experience of searching, filtering, and comparing results in order to obtain information relevant to their intended search target. Information saturation and fatigue were cited as main reasons for terminating searching. This information was often used as a resource to enhance their interactions with health care providers.

**Conclusions:**

Many participants viewed the Internet as a valuable tool for finding health information in order to support their existing health care resources. Although the Internet is a preferred source of health information, challenges persist in streamlining the search process. Content providers should continue to develop new strategies and technologies aimed at accommodating diverse populations, vocabularies, and health information needs.

## Introduction

In recent years, the quantity and quality of health information available on the Internet has increased substantially. As access to reliable, affordable, high-speed Internet access increases, the percentage of people using the Internet to search and subsequently learn from health-related information continues to grow rapidly as well. In the current climate of rising costs of health care in the United States, the role of freely available health care information is becoming more central to patients, their families and friends, and even health care providers. In order to improve the delivery of content, researchers and scientists must first develop a thorough understanding of the searching-related needs and experiences of users.

Recent studies have shed light on why and how consumers search for health information on the Internet [1-3]. In a recent 2013 survey conducted by the Pew Internet Project, 72% of respondents reported using the Internet to look for health information within the past year, with the most commonly researched topics being focused on specific diseases or conditions, treatments or procedures, and searching for doctors or other health professionals [1]. Although many people (35% of those surveyed by Pew) use the Internet to learn more about a specific symptom or medical condition they or someone else might have, clinicians and/or family and friends remain a central resource when help is needed regarding a serious health issue [2,4]. The elderly in particular are more likely to trust “living sources” of information, rather than the Internet [3]. Even among Internet users, health information is often understood in a social context. For example, 26% of Internet users reported watching or reading content related to someone else’s personal experience with a medical or health-related issue within the last 12 months [1].

Health information seeking behavior depends on a variety of factors including subjective factors (eg, intent for the search, experience in using and searching the Internet, and information preferences [5,6]) and socioeconomic factors (eg, age group, income level, education level, etc [4,7,8]). Research shows that women are more likely than men to search for health information [9] and online health consumers tend to be more educated, earn more, and have high-speed Internet access at home and at work [5,10,11]. Although low-income individuals do use the Internet, some may have difficulty distinguishing between low and high quality information [12]. Additionally, low-income disabled and homebound adults show lower rates of Internet use overall [13]. Further, our preliminary results from another study indicate that online health information seeking behavior differs significantly compared to general information searching. In particular, our data suggests that health-related queries are typically longer (ie, more words) and contextual in nature compared to general queries [14]. Also, health-related queries have higher rates of misspelled words that are typically corrected by “auto-completion” features available universally in all Web search engines such as Google and Bing [14].

There are various motivating factors for health information searching on the Internet. Aside from trying to learn more about a symptom or disorder specifically relevant to the person searching, half of online health information research is on behalf of a friend or relative [15]. Additionally, searching is often used to track specific health-related factors. For example, 60% of adults reported tracking their weight diet or exercise routine online, and 33% reported tracking specific health indicators or symptoms such as blood pressure, blood sugar, headaches, or sleep patterns [1].

A large proportion of the population uses the Internet to search for health information, and their motivations for doing so are varied [1-3]. This complex situation, along with an educationally and culturally heterogeneous population, has resulted in a barrier in the process of gathering and interpreting health information. In this context, the preferred vocabulary within and between different groups of people can differ significantly, often resulting in a variety of words being used to describe the same concept or medical condition[16-18]. Knowledge gaps can then emerge between patients and providers. One possible strategy for addressing such gaps involves developing consumer-focused vocabularies and associated infrastructure for health information retrieval that can act as an interface between parties [19]. Before such vocabularies and technologies can be developed, researchers and scientists must have a thorough understanding of the current state of online health information searching. While a large body of survey-based research has been conducted regarding this subject [1-3,20], qualitative research provides a unique perspective that can play a valuable role in informing future research and technological developments. The aim of the current study was to engage in in-depth discussions with community members about their health-related searching activities. All the study participants are residents of Olmsted County, Minnesota (MN), and are either Mayo Clinic patients, employees, or at least have one family member at home who is a patient or employee.

## Methods

### Study Participants and Recruitment

To better understand health information searching behavior and its implications for health and well-being of community members, we conducted three 90-minute focus groups of 5 to 6 individuals over the course of a 2-month span. We targeted adult, English-speaking members of the Olmsted County, MN community (where Mayo Clinic is located) and Mayo Clinic patient, employees, and family visitors. We recruited participants using flyers and online classifieds ads distributed throughout the Rochester, MN community and within Mayo Clinic. Table 1 summarizes basic characteristics of participants. Participants were provided a modest financial remuneration for participating in the study.

Moderators (JM and AK) trained in qualitative methodology facilitated discussions about the attitudes and experiences of participants related to searching for health information on the Internet. Moderators used a semi-structured moderator guide to facilitate discussion and the guide covered four major aspects: (1) participants’ perception and understanding of health care information, (2) the process of information collection on the Internet, (3) understanding and usage of information, and (4) implications of health care information for their health and well-being. Participants were asked about their thoughts and the connotations surrounding each of these themes. Oral consent was obtained from all participants. This study was approved by the Institutional Review Board at Mayo Clinic (IRB #12-005476).

Prior to participating in the focus groups, participants completed an anonymous questionnaire that included questions assessing basic demographic information and previously used sources of health information. All focus groups were audio-recorded, transcribed, and de-identified.

**Table 1:** Characteristics of patients (n=19).

Characteristic		n (%)
**Age, mean (SD; range)**		43.26 (17.0; 22-73)
**Sex**		
	Male	5 (26%)
	Female	14 (74%)
**Race**		
	White	15 (79%)
	Black or African American	0 (0%)
	Asian	4 (21%)
**Highest level of education**		
	High school or GED	0 (0%)
	Community or Jr. College	3 (16%)
	Four-year college	3 (16%)
	Graduate school	13 (68%)
**Yearly household income (US$)**		
	Less than $15,000	0 (0%)
	$15,000-$35,000	2 (11%)
	$35,001-$55,000	9 (47%)
	$55,001-$75,000	4 (21%)
	$75,001-$100,000	0 (0%)
	Over $100,000	1 (5%)
	Prefer not to answer	3 (16%)
**Prior sources used to get health information**		
	Health care providers	19 (100%)
	Family/friends	15 (79%)
	Organizations/support groups	6 (32%)
	Internet	18 (95%)
	Books/pamphlets	15 (79%)
	Other	1 (5%)
**Prior participation in research**		
	Yes	4 (21%)
	No	15 (79%)

### Data Collection and Analysis

All team members read de-identified transcripts and developed a codebook through an iterative process [21]. Using the codebook, two members of the team independently coded the transcripts in NVivo, a qualitative software application. The data were then analyzed using a grounded-theory approach (NVivo qualitative data analysis software; QSR International Pty Ltd. Version 10, 2012). Coding inconsistencies were discussed and resolved through consensus, with the input of a third team member when necessary.

## Results

### Overview

Participants candidly discussed how they used the Internet to search for health information. Through these discussions, several themes related to health motivations, content preferences, and practical applications of searching emerged. Below we summarize these data in the context of three major themes: motivations for searching, searching strategies and techniques, and information content preferences.

### Motivations for Online Health Searching

A variety of factors play a role in initiating online searches for health information. The motivations that our participants described generally fell into three main areas: (1) symptom troubleshooting, (2) searching to enhance a clinic visit, and (3) proxy searching.

Perhaps the most common motivation for everyday searching is a phenomenon that could be called “symptom troubleshooting”. With commercial online resources and other government or hospital/university-based sites that provide free, anonymous, and immediate information, many individuals’ first stop to learn more about a specific symptom is the Internet. A participant from Focus Group (FG) #3 mentioned: “For me, it was very important when I think I have a symptom, the first place I look is the Internet, especially to search for the symptoms”.

Once a particular symptom or disorder of interest is identified, participants reported that the Internet made it very easy to get more detailed information to help identify underlying causes. As a participant of FG #3 explained: “For instance if I have a pain in my foot, I am going to start looking for…information that might specify if it’s in the heel or in the toe…then I search [for] why [I have] the symptom or, if I know what I have, then I might search…to see if I can match the symptoms to that”.

Using the Internet provided a quick and easy way to troubleshoot symptoms; however, there are certain situations where using the Internet is more likely. One participant explained that the Internet is especially more convenient for superficial symptoms: “You can’tjust go find a doctor somewhere and be ‘hey, can you look at this rash on my leg’ because I hear doctors hate that” [FG #1]. The Internet provides a level of anonymity that may be helpful in situations where individuals perceive their problems to be bothersome or nuisances to doctors.

Participants often cited practical reasons related to time and money when describing their motivations for turning to the Internet for medical information or advice. One participant explained that although consulting a professional in person can be preferable, “especially when you are very concerned about your symptoms”, in other cases, as he stated, “at 9:00 at night you are not going to be able to call the doctor” [FG #3]. Another participant in FG #1 also echoed a similar sentiment: “It can’t be readily available, you may have to make a doctor’s appointment and that could take a while…and cost money and financially that might hold you back too; something that a fast care isn’t going to be able to fix”.

For non-serious medical issues, participants were generally comfortable using the Internet as a troubleshooting tool. Once a health care provider is involved, however, searching assumes a different role. In this context, participants reported using Internet searching as a means to enhance a clinic visit and be more well-prepared and well-informed during the entire health care experience with their providers. In these situations, Internet searching proved to be a valuable tool in preparing for the clinic visit. As one participant in FG #1 explained, Internet searching allowed her to walk into a surgery consultation armed with a prior understanding of possible procedures: “I specifically knew all the three main surgeries; I knew what I liked from them, what I didn’t like of them”.

This online preparation gave her the information and ability to “say what about this, what about that, why are we doing this, why are we doing that?” [FG #1]. Participants agreed that such preparation facilitates “a more enriched experience” [FG #1] and allows patients to “become more knowledgeable” and “ask better questions” to providers [FG #2]. This participant goes on to explain how such a dynamic increases communication and education and “builds the patient/provider relationship”; “If you are taking an interest in what it is you have and asking the kind of questions that allow them to further educate you, I think that shows a real interest” [FG #2].

Another participant expanded on this idea and explained how an enriched patient/provider relationship involves more than developing a healthy rapport and can actually improve health outcomes in certain situations: “I mean my mom had a weird thyroid thing and she was all over the Internet, and still is, but she would bring stuff to her doctor and she actually like did solve some mysterious things and she gave stuff to her doctor and her doctor I think is a great doctor but there is so much information and the doctors don’t get it all” [FG #2].

In the previous example, the participant’s mother used the Internet for two of the main motivations that emerged from our focus groups: to troubleshoot a thyroid condition and to enhance her visits with her doctors. Although this participant’s mother was able to do the searching and advocating on her own, many participants had parents, grandparents, or other family members who were not as comfortable or capable. These situations highlight the third main motivation for searching that our participants discussed: searching for someone else, or proxy searching. All of the focus groups had participants who reported searching on behalf of someone else. For many, it was a frequent occurrence.

Computer literacy was often cited as a main reason for proxy searching, as many participants had relatives who were “afraid of using it [computers and the Internet]” [FG #1]. However, proxy searching was also a useful tactic when the individual searching sought to protect their relative from additional emotional burdens, even when the relative was computer literate. One focus group participant explained: “Well, I have done searches for my parents before…When I looked up stuff [about] breast cancer on the Internet, [I told them] do not look it up because you’re going to be scared. As a third person, even though she is my mom, I know how to decide and to remove myself from the situation, but she is not going to be able to do that” [FG #3].

### Searching Strategies and Techniques

In terms of the actual mechanics of searching, participants described using a common set of steps and procedures that began with commonly used search engines, continued to shop around for information from various sources, and ended with information saturation and exhaustion.

Regardless of the underlying motivations for searching, almost all searches shared a common starting point from an online Web search engine: Google. Ease of use*—*“you can ask the most stupidest questions and have a pretty good shot of getting an answer” [FG #1]—and quality of results—“[Google] brings up the most variety of answers” [FG #1]—were the primary reasons for choosing Google cited by our participants.

Although Google is by far the most common first step to searching, its main use is simply as a tool to reach other sites. One participant mentioned: “Google’s just a way to get there” [FG #1]. Another participant expanded on this view, adding “I agree. I am not putting my trust in Google; I am only putting my trust that Google is going to give me a variety. My trust is actually embedded only in the searches I click, it is just the outlet to get me there, it is just the bridge” [FG #1].

Once Google supplied a list of relevant sites to visit, most participants reported visiting many sites in order to satisfy their searching demands. This technique allows participants to “shop around and have multiple sources” without having to use exact phrasing [FG #1]. The information shopping process described by participants often included multiple side-by-side comparisons. One participant mentioned: “Because you can multiple open window task bars and tabs on the Web browser, I open every single one on the first page in each of the task bars and compare all of them” [FG #2].

This technique facilitated the information shopping experience and gives greater confidence in results because “you get as much information as you can if [all the websites] have the same information” [FG #2]*.* Many participants used the tabs function of Web browsers to compare multiple websites at once.

Participants described a common sequence of events that led to the termination of the search process. As the comparing and filtering process of multiple websites progresses, participants reported that eventually “all the information is basically the same” [FG #1]. Although another participant acknowledged that “there are always additional links to go to” [FG #1], other participants explained that once results became irrelevant to their original search query it was time to stop the search process. One participant explained: “If you go down to the 17th, 20th, 30th option under Google, you find that what you are looking for is the 30th degree of separation. It is just not as relevant to what it is you are trying to research anymore” [FG #1].

Some participants also reported a sense of being “lost” or “completely forgetting where you started”, especially in cases of performing broad searches. The resulting confusion can lead to becoming “unmotivated” to continue searching, even if the original query has not been resolved [FG #1].

In addition to information saturation, subjective fatigue was an indicator participants described as a reason for ending the search process. After a long, drawn-out search process, participants reported getting “tired with the screens” and feeling “exhausted” [FG #1]. Another participant compared the process to shopping: “If you know what you want, you can go to ten different places to try to find that one thing, but after a while…you are going to be hitting your head against the wall…it gets exhausting” [FG #1].

Ultimately, the participants described searching for health-related information as a rigorous process of comparing and contrasting various sources against personalized criteria based on need and individual appraisal of reputation. This filtering process generally continues until the results become repetitive and/or the searcher becomes fatigued.

### Content Preferences

Major search engines can easily produce thousands of results for any given query. How then do patients and consumers select which websites to gather health-related information? Although every search is unique, participants overwhelmingly preferred sites based on two main factors: reputation and advertising (or lack thereof).

Participants often commented that they “tend to go for the sites that are most reputable” [FG #1]. While the importance of reputation applied to all websites, regardless if they were related to health, participants also reported placing a higher standard of quality on health-related information. As one participant explained, “Health is unlike any other consumer type of website…I take it to a totally different level. I want to have the best, you only have one body” [FG #1]. Making sure they had “the best” gave participants comfort in knowing they were receiving accurate information. Often “the best” is synonymous with dealing with a “reputable institution”, which is in turn largely influenced by branding. One participant explained: “When you are dealing with a company, an organization that has a good reputation, then you feel more confident that you are getting the right information” [FG #3].

In addition to pure name recognition, participants reported that institutions “earn trust…through publications, research, and education” [FG #2]. Additionally, “how [websites or institutions] are ranked” or if they are “well known” contributed to participants’ conception of reputation [FG #2]. Finally, participants were more likely to view sources of health information as reputable if they were domestic. As one participant explained, “I would rely more heavily on those [domestic] institutions than a foreign hospital that may be quite good but is somewhere outside of the United States” [FG #2].

While reputation played a major role in determining which websites to trust for our participants, advertising and commercial interests often dissuaded them. Almost all of our participants reported avoiding websites that had visible advertising or were obviously profit-oriented. As one participant explained, “If I see ads, I question the motivation for providing information that they have” [FG #1]*.* Another participant explained the aversion in the context of a wider trend of commercialization of medicine: “I think for me it scares me how, and I suppose this could go onto a variety of different things, but it scares me how medicine has transformed into such a consumer-driven place” [FG #1].

Most of our participants shared distaste for commercial interests in their searching behavior; however, in some cases it had more to do with the perception of profit-driven motivations rather than the true nature of the business or organization. In response to a question regarding whether or not participants thought that MayoClinic.com, the commercial consumer health information portal owned and maintained by Mayo Clinic, was a “commercial” website, one participant responded, “Well, you don’t see a lot of advertising on the Mayo site…I don’t see a lot going on the sides all the way down the page flashing at me, I don’t have a lot of popups that come at me” [FG #1].

Although Mayo Clinic does indeed utilize advertising on the website, the combined name recognition, familiarity, and subtle nature of advertisements was enough to retain credibility for many of our participants. We acknowledge that there might be an inherent bias in this finding since the study participants were either Mayo Clinic patients, employees, or at least have one family member at home who is a patient or employee.

## Discussion

### Principal Findings

Our goal in collecting these qualitative data was to better understand how consumers use and search for health information on the Internet to inform the development of more personalized health information searching and delivery applications. The participants in this study described a common experience of searching for health information that largely mirrors recent large-scale survey data. Most of our participants see the Internet as a potentially valuable tool to find information about health and medical conditions; yet, they did point to the challenge of efficiently addressing their particular needs given the vast amounts of information. This reflects the challenge of streamlining and personalizing information for a user base that is diverse both in terms of individual background and need. The data presented here, particularly in the context of content preferences and searching techniques, may be beneficial to researchers and content providers as they develop new strategies for delivering health information.

Many participants shared examples of how they use information they found through Internet searches in their efforts to enhance their interactions with their health care providers. Examining these data in the context of increasing health costs and physician time constraints provides valuable insight into the challenges and opportunities consumers and physicians will encounter in years ahead. Many of our participants reported using Internet health searching as a means of enhancing clinic visits, either through preparation or post-appointment follow-up. Some concerns exist regarding how doctors may react to patients introducing health information gathered from the Internet into the exam room, and indeed previous research has indicated that some physicians view such occurrences negatively [22,23]. Patients, on the other hand, tend to view Internet health searching as an additional resource to complement the still highly valued patient/physician relationship [24,25]. Our data also support this view of the patient perspective, as our participants viewed online health searching as a means to “build the doctor-patient relationship” [FG #2]. How physicians respond likely depends on physician communication skills and whether or not the physician feels challenged [26]. The participant experiences and opinions described here are largely from a patient perspective and are largely positive in the context of using health information from the Internet to enhance visits. These perspectives may be useful in framing future research focused on physician perspectives on using such information in office visits.

Recently, the amount of time doctors spend in front of patients has received attention in the media [27,28]. Having patients armed with information and questions prior to office visits may help improve care in the current realities of decreased face time with doctors, which today can be as low as 8 minutes on average [28]. This of course necessitates that the information patients gather be of high quality. Indeed, research suggests the quality of information that patients present ultimately determines its effect on the patient/physician relationship; while accurate information can be helpful, inaccurate information may be harmful [29]. Our future work will therefore focus on ways to develop consumer health information technology solutions to facilitate the transmission of accurate, trustworthy, validated information to consumers to ensure that online health information searching enhances, rather than hinders, care.

### Limitations

This study contained a few important limitations. Due to recruitment constraints, our study population was limited to adults within Olmsted County, MN. All participants were either employees or were family members of employees and patients at Mayo Clinic, where the study took place. Additionally, our sample was highly educated, with all participants having attained at least a community college degree, and 68% having completed graduate school. We were therefore unable to explore the perspectives of a more diverse population. It is also important to consider our choice of study design when interpreting the data we presented. In this study, we used qualitative approaches such as grounded theory and focus groups method for data collection and analysis. These qualitative methods allow us to contextualize participants’ understandings and experiences, to track variations in how concepts are understood, and to uncover novel findings that may warrant further investigation [30]. In this way, we are able to make, as Giacomini and Cook describe, an “empirically-based contribution to ongoing dialogue” [31]. The overarching goal of qualitative research is to explore and describe *particularities* of a social phenomenon rather than producing generalizable results. But, findings from a small sample size in a qualitative research can help developing hypothesis for a quantitative study to produce generalizable findings from a larger sample size. Our study participants were recruited from a limited subset of individuals that was readily accessible in a community dominated by the health care industry. In doing so, our goal is not to present data that can or should be generalized to a wider population, but rather to explore pertinent issues with a level of depth that is not possible with standard quantitative (and generalizable) methodologies. Indeed, we cannot claim that the experiences described here are representative of all Internet users; however, they can inform the development of future work and research in areas of streamlining content delivery and patient/physician interaction.

### Conclusions

We conducted this qualitative study to gain a deeper understanding of search behavior in order to inform future technological developments in personalizing online information searching and content delivery. Although the Internet was a preferred source of health information for almost all of our participants, from a consumer and patient perspective challenges persist in streamlining the process of identifying reliable and high quality content that also matches the intended search target of the user. Our participants described a current search paradigm consisting of drawn-out user-driven comparisons of content obtained from multiple sources of varying quality and unverified validity. As consumers continue to use information gathered from the Internet to enhance their interactions with health care providers, new strategies for delivering health information on the Internet must be developed that accommodate diverse backgrounds and clinical needs.
